# Pre-implant right ventricular function might be an important predictor of the response to cardiac resynchronization therapy

**DOI:** 10.1186/1476-7120-9-28

**Published:** 2011-10-26

**Authors:** Magnus Edner, Margareta Ring, Peter Henriksson

**Affiliations:** 1 Karolinska Institutet, Division of Cardiovascular Medicine, Department of Clinical Sciences, Danderyd Hospital, Stockholm, Sweden; 2 Department of Molecular Medicine and Surgery, Karolinska Institutet, Stockholm, Sweden

## Abstract

**Objective:**

Cardiac resynchronization therapy is proven efficacious in patients with heart failure (HF). Presence of biventricular HF is associated with a worse prognosis than having only left ventricular (LV) HF and pacing might deteriorate heart function. The aim of the study was to assess a possible significance of right ventricular (RV) pre-implant systolic function to predict response to CRT.

**Design:**

We studied 22 HF-patients aged 72 ± 11 years, QRS-duration 155 ± 20 ms and with an LV ejection fraction (EF) of 26 ± 6% before and four weeks after receiving a CRT-device.

**Results:**

There were no changes in LV diameters or end systolic volume (ESV) during the study. However, end diastolic volume (EDV) decreased from 226 ± 71 to 211 ± 64 ml (p = 0.02) and systolic maximal velocities (SMV) increased from 2.2 ± 0.4 to 2.6 ± 0.9 cm/s (p = 0.04). Pre-implant RV-SMV (6.2 ± 2.6 cm/s) predicted postoperative increase in LV contractility, p = 0.032.

**Conclusions:**

Pre-implant decreased RV systolic function might be an important way to predict a poor response to CRT implicating that other treatments should be considered. Furthermore we found that 3D- echocardiography and Tissue Doppler Imaging were feasible to detect short-term changes in LV function.

## Background

Chronic heart failure (CHF) is a frequent cause of in-hospital health care with a high cost. These patients have a poor quality of life (QoL) and a high readmission rate despite drugs such as RAAS- and beta-blockers, diuretics and aldosterone-antagonists which all have positive prognostic effects [1-5].

Cardiac Resynchronization Therapy (CRT) is established as an effective therapy to reduce mortality and morbidity in patients with systolic heart failure (HF) and prolonged QRS in functional class (NYHA) III-IV despite optimal medical treatment [6-10].

Dyssynchrony was pointed out as a baseline predictor of a beneficial effect of CRT in an analysis of the PROSPECT trial [11,12]. The analysis also pointed out other important baseline predictors of a beneficial response to CRT such as female gender, non-ischemic ethiology, NYHA-class III, QRS-duration and no history of ventricular tachycardia. NYHA-class IV was not associated with CRT response and one reason might be that right ventricular (RV) apical pacing sometimes deteriorates LV function [13,14] and many NYHA-class IV HF patients have RV dysfunction. The pathophysiologic explanations are not known for sure but could, at least partly, be that an already injured RV cannot manage the increased preload caused by better LV function in some patients. In the MADIT II trial it was found that patients receiving RV pacing had an increased prevalence of worsened heart failure than those with less RV pacing (15).

## Aims

We assessed whether pre-implant systolic RV function could predict short-term response to CRT and if 3D- and TDI-echocardiography could be used to detect early changes in LV dimensions and function.

## Materials and methods

### Study Population

We enrolled 22 consecutive HF patients fulfilling standard criteria for CRT. All patients had a reduced systolic left ventricular (LV) function with an ejection fraction (EF) below 35%, QRS - duration > 120 ms and NYHA class III-IV despite optimal medical treatment (table 1).

**Table 1:** Baseline characteristics (n = 22)

Variable	HF patients
Age (years)	72 ± 11 (47-88)
Gender (male)	20
Heart Rate, bpm QRS-width, ms	69 ± 18 (45-129) 155 ± 20 (120-190)
Function Class, NYHA (n)	
III	20
IV	2
Previous Myocardial Infarction	11
Hypertension	3
Dilated Cardiomyopathy	4
Diabetes	3
ACE/ARB	22
Diuretics	22
Beta-blockers	19
Spironolactone	15

Mean ± SD unless otherwise stated. HF = heart failure, NYHA = New York Heart Association,

ACEi = Angiotensin Converter Enzyme Inhibitor, ARB = Angiotensin Receptor Blocker.

Exclusion criterion was atrial fibrillation.

All patients gave informed consent. The study adhered to the Helsinki declaration and was approved by the Regional Ethical Review Board.

### Echocardiography and Tissue Doppler Imaging

The patients were examined in the left lateral position and Tissue Doppler Imaging (TDI) echocardiography was acquired in three apical views using the Vivid 7 or 5 (the later for 3-D echocardiography only) systems (Vingmed, Hortem, Norway), with a 2.5 MHz probe. Three consecutive beats were registered and mean values were used for further analysis. Analyses were made off-line (Echo-Pac software, Hortem, Norway). The 16 segments LV-model of the American Society of Echocardiography was used for orientation [16]. Base and mid LV-segments, a total of 12, were used together with base and mid RV-segments in the 4-chamber view. The LV end diastolic dimension (LVEDd) was measured in the parasternal long axis (PLAX) view from 2D registrations. The LV end systolic diameter (LVESd) was measured in the same view with the smallest achievable LV size. Regional systolic contractility was assessed as systolic maximal velocity (SMV) during the ejection phase in the same segments. Tissue tracking (TT) was used to measure the longitudinal function in each segment. A mean value for 12 LV segments was calculated to achieve a measurement of global contractility.

Trans thoracic 3D Echo was performed using a 2.5 MHz transducer mounted in a handheld rotation device. The cardiac images were recorded from the apex during end-expiratory apnoea within one breath hold, whereby the need for respiratory triggering was abolished as previously described [17]. End diastolic volume (EDV) and End systolic volume (ESV) were measured and ejection fraction (EF) was calculated by the formula EDV-ESV/EDV × 100. All examinations were performed before and four weeks after receiving the CRT-device and stored for later off-line analysis. Volumes measured by 3D echo are almost as sensitive as measured by Magnetic Resonance Imaging. Thus, we used a combination of echo methods since we believe that they are enough sensitive to detect changes during short-term follow-up.

### Biventricular Pacemaker Implantation

All patients received a CRT device (Insync III ^®^, Medtronic) implanted transvenously as described previously [17].

## Statistical analyses

Continuous variables with a normal distribution were analyzed by paired Students t-test and given as mean ± SD. Other variables were analyzed by nonparametric tests and data are given as medians and range. All analyses were performed by Statistica^® ^version 9.1 software (StatSoft, Inc., Tulsa, USA). Multivariable regression was performed by the best subset technique of Statistica. The ability of the right ventricular function variables to explain change in left ventricular function was the main analysis. The robustness of the variables to predict this change was also tested by using variable importance (VIP) in a Projection to Latent Structures (PLS)-regression analysis. We regarded p < 0·05 (two-sided) as significant.

## Results

Baseline characteristics of the patients are presented in table 1. Clinical and conventional, 3D and TDI echocardiographic changes from before to four weeks after CRT implantation are shown in table 2.

**Table 2:** Clinical and Echocardiographic (conventional, 3D and TDI) changes from pre-implantation to four weeks after CRT implantation

Variable	Pre- implantation	Four weeks after CRT-implantation	p-value
QRS, ms	155 ± 20	162 ± 29	n.s.

NT-proBNP, pg/ml	2925 ± 2611	3290 ± 3545	n.s.

Minnesota-score, median(range)	49 (11-90)	30 (1-69)	0.0004

NYHA-class, median(range)	3 (2-4)	2 (1-3)	0.0064

LV EDd, mm	67.6 ± 9.2	67.4 ± 8.9	n.s.

LV ESd, mm	59.2 ± 10.1	59.3 ± 9.9	n.s.

MR, 0-4, median(range)	1.5(0.5-2.5)	1.0(0.0-2.5)	0.063

LV EDV, ml	226 ± 71	211 ± 64	0.020

LV ESV, ml	169 ± 64	163 ± 59	n.s.

EF, %	24 ± 7	24 ± 8	n.s.

Ts septum-lateral, ms	60 ± 31	52 ± 44	n.s.

LV TT, mm	3.1 ± 1.2	3.9 ± 1.5	0.038

LV SMV, cm/s	2.2 ± 0.4	2.6 ± 0.9	0.045

RV TT, mm	8.8 ± 4.8	9.4 ± 3.7	n.s.

RV SMV, cm/s	6.2 ± 2.6	6.5 ± 2.6	n.s.

Mean ± SD unless otherwise stated. MR = mitral regurgitation, Ts = dyssynchrony, SMV = systolic maximal velocity, LV EDd = left ventricular end diastolic diameter, LV ESd = left ventricular end systolic diameter, EF = ejection fraction. EDV = end diastolic volume, ESV = end systolic volume, TT = tissue tracking, RV = right ventricular.

There were no changes in LV diameters or ESV. However, EDV decreased from 226 ± 71 to 211 ± 64 ml (p = 0.02) and SMV increased from 2.2 ± 0.4 to 2.6 ± 0.9 cm/s (p = 0.04*)*. The increase in LV-SMV could by 41% (adjusted R^2 ^in best subset multiple regression) be explained by pre-implant RV-SMV and RV-TT. These two variables were also shown to be the most important predictor variables in a PLS regression. Inclusion of pre-implant LV-SM and LV-TT in the multivariable regression did not improve the explanation of the change in LV-SM (Table 3). The significance of RV-SM persisted but RV-TT was no longer significant.

**Table 3:** Results of multivariable regression of the influence of pre-implant RV-SM, RV-TT, LV-SM and LV-TT on change in LV-SM four weeks after CRT implantation

	Parameter	95.00% Confidence interval	p-value	Beta-value
Intercept	0.067	(-1.74-1.87)	0.94	

RV SM	0.25	(0.024-0.47)	0.032	0.85

RV TT	-0.13	(-0.31-0.05)	0.15	-0.59

LV SMV	-0.18	(-1.46-1.10)	0.77	-0.10

LV TT	-0.01	(-0.53-0.51)	0.97	-0.014

RV = right ventricular, LV = left ventricular, SMV = systolic maximal velocity, TT = tissue tracking.

## Discussion

We found that pre-implant RV systolic function as assessed by TDI could predict short-term response to CRT. The results are plausible from a mechanistic point of view but should of course be reproduced in a larger cohort of patients and with a longer follow-up.

The clinical implication of a role of pre-implant systolic RV function in the response to CRT should of course be that other treatments than CRT should be considered in patients with severe RV HF.

Our findings are supported by others using other methods and longer follow-up to study the importance of pre-implant RV systolic function [18,19]. Scuteri et al used an extensive echo-Doppler examination and found that preimplant M-mode (TAPSE), RV systolic pressure measured by Doppler, RV dimensions and RV area change to be related with post-implant LV ESV remodelling [18]. They also found these relations to be consistent independent of aetiology and degree of dyssynchrony indicating the important roll of the pre-implant RV function. They suggest TAPSE < 14 mm, a simple and highly reproducible method, as cut-off value to define advanced RV dysfunction and found this cut-off to be associated with a twofold risk of the combined end point of death and emergency heart transplantation. In another study by Tabereaux et al a visual grading by 2D echocardiography of the RV function was used [19]. RV EF < 40% was the definition of RV systolic dysfunction and found to be associated to less response to CRT during 6 month's follow-up. Thus, the importance of preserved pre-implant systolic RV function has been demonstrated by different methods in HF patients with standard indication for CRT.

A suggested echocardiographic cut-off value of response to CRT is an decrease by approximately 10% in the end diastolic and end systolic volume [20,21]. However, this is after 12 weeks CRT and there are no certain parameters to be measured to define a response already after four weeks of CRT. Thus, in this study we chose a combination of clinical parameters such as patient-assessed functional class and quality of life and echocardiographic parameters such as LV diameters (conventional echo), LV volumes (3D echo) and myocardial velocities and longitudinal movement (TDI) to decide whether there where a response after four weeks of CRT or not. According to this approach 68% of the patients responded to CRT in this study, which main aim however was to assess the importance of the pre-implant systolic RV function. 3D echo has been reported almost as sensitive as magnetic resonance imaging when measuring LV volumes and Müller-Brunotte at al reported TDI to be more sensitive than conventional echo measuring effects after treatment of hypertension induced LV hypertrophy [22]. It seems obvious that echocardiography should be used more when planning or doing follow-up in CRT since a lot of important information is given. However, the technique requires time and special interest and if it has to be done in all patients it might result in underuse of the CRT, a very effective treatment which probably is underused already today.

One explanation to the suggested importance of pre-implant systolic RV function might be a preload increase caused either by an acute deterioration in RV systolic function or by a miss match improvement where the LV function improves more than the RV function resulting in non-responding to CRT.

Furthermore, in the revised ESC guidelines on device therapy in HF it is pointed out that conventional pacing might increase symptoms and deteriorate LV function and therefore biventricular pacing is recommended regardless of QRS prolongation in patients with LV dysfunction and signs of HF [23].

In conclusion our findings implicate that pre-implant RV systolic function might be an important predictor of the poor response to CRT in some patients with biventricular heart failure and other treatments should be considered in these patients. Furthermore 3D- echocardiography and TDI seem feasible to detect changes in LV function during short-term follow-up.

## Disclosures

No conflicts of interest.

## Authors' contributions

ME was responsible for planning and writing. MR and PH performed echocardiographic and statistical analyses and helped writing manuscript. All authors read and approved the final manuscript.
